# Dizziness, Lower Limb Weakness, and Falls in COPD: A Prospective Longitudinal Pilot Study Using Monthly Home Diaries

**DOI:** 10.1002/pri.70363

**Published:** 2026-10-22

**Authors:** Danielle Ferreira de Santana Silva, Bianca Maria Santos Alcântara, Jefferson Ricardo Santos da Silva, Rafaela Pedrosa, Eduardo Eriko Tenório de França, José Heriston de Morais Lima

**Affiliations:** ^1^ Graduate Program in Physical Therapy Federal University of Paraíba (UFPB) João Pessoa Paraíba Brazil; ^2^ Undergraduate Program in Physical Therapy Federal University of Paraíba (UFPB) João Pessoa Paraíba Brazil; ^3^ Postgraduate in Software Engineering and Quality University Center of João Pessoa (UNIPÊ) João Pessoa Paraíba Brazil

**Keywords:** accidental falls, chronic obstructive, dizziness, muscle weakness, physical therapy, pulmonary disease

## Abstract

**Background and Purpose:**

Falls affect approximately half of individuals with chronic obstructive pulmonary disease (COPD) and are associated with hospitalization, accelerated functional decline, and early institutionalization. Existing evidence suggests that single‐visit assessments have limited and inconsistent ability to predict the future falls in individuals with COPD, highlighting the need for alternative longitudinal approaches. To our knowledge, this is the first prospective study to explore the longitudinal association between diary‐recorded symptoms and falls in COPD.

**Methods:**

Prospective observational cohort pilot study (*n* = 27), with 6‐month follow‐up. Falls and seven symptom variables were recorded monthly via structured home diaries, complemented by a standardised monthly telephone/messaging follow‐up. Receiver operating characteristic (ROC) curves with 95% bootstrap confidence intervals (CI) and optimal cut‐off points using the Youden index were calculated, with internal validation by optimism‐correction bootstrap. Mann–Whitney U tests compared groups (*α* = 0.05).

**Results:**

Thirteen patients (48.1%) experienced at least one fall. Dizziness showed the strongest discrimination in this sample (area under the ROC curve [AUC] = 0.813; optimism‐corrected 0.81; *p* = 0.002; optimal cut‐off ≥ 1 month; specificity 85.7%; positive likelihood ratio [+LR] 4.84). Lower limb weakness for ≥ 2 months was the most sensitive discriminating variable (AUC = 0.739; *p* = 0.034; sensitivity 92.3%). Among baseline physiological measures, only the FEV1/FVC ratio showed acceptable discrimination (AUC = 0.734; *p* = 0.041), in a paradoxical direction (higher among fallers); Timed Up and Go, maximal inspiratory pressure, and quadriceps ultrasound did not significantly discriminate fallers from non‐fallers.

**Discussion:**

Longitudinal symptom monitoring via monthly home diaries showed higher apparent discrimination than single‐visit baseline assessments in this pilot sample. These findings are exploratory and hypothesis‐generating, supporting a confirmatory study with an adequate sample size. Monthly diary monitoring is low‐cost, feasible by telephone, and may represent a complementary approach to fall risk assessment in COPD.

**Trial Registration:**

Registered on ClinicalTrials.gov, identifier NCT06827379 (PI: Eduardo Eriko Tenório de França)

## 1 Introduction

Chronic obstructive pulmonary disease (COPD) is a systemic condition whose consequences extend far beyond airflow limitation. Progressive skeletal muscle dysfunction and autonomic dysregulation converge to compromise postural stability and fall‐prevention reflexes, placing individuals with COPD at a substantially elevated risk of falls compared with age‐matched individuals without lung disease (Global Initiative for Chronic Obstructive Lung Disease (GOLD) 2025). Epidemiological evidence consistently indicates that 30%–50% of individuals with COPD living in the community experience at least one fall during a 6‐month observation period, a rate two to three times higher than in age‐matched controls (Beauchamp et al. 2012; Roig et al. 2011).

The consequences of falls in this population are disproportionately severe. Each fall event multiplies the risk of hospitalization fivefold to eightfold, triggers fear of falling that further restricts physical activity, and is strongly associated with accelerated functional decline and early institutionalization (i.e., an anticipated need for formal assisted care or transfer to a supervised living environment resulting from loss of functional independence) (Oliveira et al. 2021, 2015). In a disease already characterized by progressive functional disability (Global Initiative for Chronic Obstructive Lung Disease (GOLD) 2025; Oliveira et al. 2021), falls represent a largely preventable amplifier of functional loss.

The main clinical challenge is that falls are difficult to predict using conventional assessment approaches. Prospective studies have attempted to identify baseline predictors, including the Timed Up and Go (TUG) test, grip strength, Berg Balance Scale, and spirometric severity, and have found that none consistently discriminates individuals with COPD who will fall from those who will not (Beauchamp et al. 2012; Roig et al. 2011; Hakamy et al. 2018). The conceptual limitation of these approaches is their reliance on cross‐sectional single‐visit assessments to predict a dynamic and fluctuating outcome. Existing fall‐risk assessment in COPD typically combines functional testing, medication review, balance training, and pulmonary rehabilitation, but lacks a longitudinal component.

Two symptoms are mechanistically associated with precipitating factors for falls in COPD: dizziness and lower limb weakness. Dizziness arises from exercise‐induced arterial oxygen desaturation, autonomic dysfunction, and adverse effects of widely prescribed medications, including beta‐blockers, diuretics, and antihypertensives (Menant et al. 2012; Tinetti et al. 2000). Lower limb weakness, a clinical expression of peripheral myopathy, impairs reactive muscle corrections that prevent falls during postural transitions (Beauchamp et al. 2012; Robles et al. 2011). Crucially, both symptoms fluctuate over time with disease activity, medication adjustments, and exacerbation cycles (Menant et al. 2012; Tinetti et al. 2000), making them natural longitudinal phenomena.

Monthly home diaries have been validated primarily for the detection of exacerbations and monitoring of symptom burden in COPD specifically (Seemungal et al. 1998; Wedzicha et al. 2017). Whether extending diary monitoring to dizziness and lower limb weakness may generate clinically useful fall‐risk signals remains unknown. Monthly home diaries are inherently subject to recall bias and depend on sustained participant adherence; nonetheless, they remain one of the few feasible strategies for capturing symptom fluctuation between clinical visits, and were therefore chosen as the primary monitoring method in this pilot study. Additional symptoms recorded in the diary (malaise, extra breathlessness, difficulty rising from a chair, difficulty walking, and difficulty sleeping) were selected based on their plausible impact on mobility, balance, and fall risk, and are commonly reported during COPD symptom fluctuation and exacerbation.

The aim of this study was to investigate the occurrence of dizziness, lower limb weakness, and other diary‐recorded symptoms in relation to falls in individuals with COPD, prospectively recorded through monthly home diaries over 6 months, and to define exploratory clinical cut‐off values using ROC analysis with bootstrap confidence intervals.

## 2 Methods

### 2.1 Study Design

This was a prospective longitudinal pilot study conducted at a university‐affiliated cardiorespiratory physical therapy research laboratory [institution and laboratory names blinded for review] in Brazil. This pilot substudy was nested within a parent project on inspiratory muscle training in COPD [funding call and process number blinded for review], which explains the concurrent respiratory‐muscle and ultrasound assessments. Participants did not receive inspiratory muscle training or any other intervention during the 6‐month observational follow‐up reported here; during this period, they were monitored purely observationally, through monthly telephone contact in which a structured questionnaire (Google Forms) was administered to ascertain respiratory exacerbation symptoms and the occurrence of falls, with no exposure that differed systematically between participants. Recruitment took place from September 2025 to March 2026, with a 6‐month follow‐up for each participant. A 6‐month duration was chosen because this study was designed as a pilot investigation aimed at evaluating feasibility, estimating effect sizes, and generating parameters for sample‐size calculation in a future confirmatory study (Table S1); a 12‐month follow‐up is planned for that confirmatory study. This report follows the STROBE statement for cohort studies (Von et al. 2007).

### 2.2 Subjects

Inclusion criteria were: confirmed COPD diagnosis (post‐bronchodilator ratio of forced expiratory volume in 1 second to forced vital capacity [FEV1/FVC] < 0.70 per GOLD criteria [1]), age 40–75 years, and clinical stability at enrolment, defined as the absence of a physician‐diagnosed acute exacerbation (per GOLD criteria) in the preceding month, confirmed through medical records and participant self‐report. Exclusion criteria were: functional disability precluding study assessments, progressive neurological disorders, cognitive impairment, active malignancy, chronic pain unrelated to COPD, or acute exacerbation in the month prior to recruitment. Sociodemographic data (age, sex, marital status, education, and occupation) and clinical data were collected at baseline using a standardised semi‐structured form.

Potential confounders identified a priori, including polypharmacy (particularly vestibulotoxic agents, antihypertensives, and sedatives), prior fall history, orthostatic hypotension, vestibular disorders, peripheral neuropathy, and visual impairment, were recorded at baseline where feasible and are summarised in Table 1. As detailed in Statistical Analysis, these factors could not be statistically controlled given the pilot sample size.

**TABLE 1:** Sample characteristics and comparison between fallers and non‐fallers (*n* = 27).

Variable	Total (*n* = 27)	Fallers (*n* = 13)	Non‐fallers (*n* = 14)	Effect size	*p*
Age (years)	65.5 ± 9.4	65.8 ± 10.2	65.3 ± 8.9	*r* = 0.038	0.884
Sex, male, *n* (%)	15 (55.6)	7 (53.8)	8 (57.1)	OR = 0.88	1.000
BMI (kg/m^2^)	25.7 ± 5.2	26.6 ± 4.4	24.9 ± 5.9	*r* = 0.220	0.344
MRC scale (median, IQR)	2 (1–3)	2.0 ± 1.5	2.0 ± 1.2	*r* = −0.027	0.919
FEV1% predicted	48.5 ± 19.9	53.8 ± 21.3	43.6 ± 17.8	*r* = 0.297	0.198
FEV1/FVC	0.57 ± 0.11	0.61 ± 0.09	0.53 ± 0.10	*r* = 0.467	**0.041** *
GOLD 1–2, *n* (%)	19 (70.4)	11 (84.6)	8 (57.1)	OR = 4.13	0.209
TUG, mean (s)	9.78 ± 2.53	9.52 ± 2.75	10.03 ± 2.39	*r* = −0.154	0.512
MIP (cmH_2_O)	54.9 ± 22.1	56.6 ± 25.9	53.3 ± 18.8	*r* = 0.033	0.903
RF echo intensity (AU)	75.7 ± 18.7	75.7 ± 16.2	75.7 ± 21.4	*r* = −0.044	0.865
VI echo intensity (AU)	68.7 ± 22.9	66.5 ± 17.9	70.7 ± 27.3	*r* = 0.000	1.000
VI thickness (mm)	7.90 ± 3.62	8.79 ± 2.70	7.07 ± 4.24	*r* = 0.346	0.132

*Note:* Mean ± SD unless noted. Mann–Whitney U or Fisher's exact test. Effect size: rank‐biserial correlation (*r*) for continuous variables (positive values indicate higher values among fallers); odds ratio (OR) for categorical variables. Bold values indicate statistically significant differences between groups (*p* < 0.05).

Abbreviations: AU, arbitrary units; BMI, body mass index; MRC, Medical Research Council; TUG, Timed Up and Go; MIP, maximal inspiratory pressure; RF, rectus femoris; VI, vastus intermedius.

^*^ *p* < 0.05.

### 2.3 Procedure

#### 2.3.1 Fall Ascertainment

Falls were defined as any unintentional descent to the ground or a lower surface (Bull 1987), or any loss of balance requiring support to avoid falling (Zecevic et al. 2006), not attributable to a violent blow, loss of consciousness, or sudden paralysis. Each participant received a structured monthly diary at enrolment, with fields for date, location, circumstances, and consequences of each fall. The primary outcome was the occurrence of ≥ 1 fall during 6 months (binary: fallers vs. non‐fallers). The secondary outcome was recurrent falls (≥ 2 falls). Both event types (completed descents and balance losses requiring support) were captured under this single definition and recorded in the diary and in the study database as a single aggregated fall count per participant, with no field distinguishing completed falls from near‐falls; consequently, the two event types cannot be disaggregated in the present analysis, and the reported fall counts and derived outcomes should be interpreted as reflecting this combined definition.

#### 2.3.2 Longitudinal Symptom Diary

Participants recorded the occurrence of each symptom prospectively in their home diary as events occurred throughout each month. To support adherence and standardise data capture, a trained physiotherapist additionally contacted each participant once a month (by telephone) and administered a standardised semi‐structured questionnaire (Google Forms) to confirm and consolidate the events recorded for that month, for seven symptoms: (Global Initiative for Chronic Obstructive Lung Disease (GOLD) 2025) dizziness or vertigo; (Beauchamp et al. 2012) lower limb weakness; (Roig et al. 2011) difficulty rising from a chair; (Oliveira et al. 2021) difficulty walking; (Oliveira et al. 2015) feeling generally unwell; (Hakamy et al. 2018) extra breathlessness beyond usual; and (Menant et al. 2012) difficulty sleeping. Each variable ranged from 0 to 6 months. Symptoms were standardised using a reference card provided at enrolment (Supporting Information S2). The card operationalised each symptom in plain language; to our knowledge, no COPD‐specific validated definitions exist for these items, which were therefore treated as pragmatic, study‐specific descriptors. This design combined prospective self‐recording with structured monthly verification; because consolidation occurred at monthly intervals, some recall over the preceding weeks cannot be excluded and is addressed in the Limitations.

#### 2.3.3 Baseline Assessments

Spirometry (FEV1% predicted, FEV1/FVC) was performed according to ATS/ERS standards (Graham et al. 2019); disease severity was classified per GOLD stage (Global Initiative for Chronic Obstructive Lung Disease (GOLD) 2025). Comorbidities and medications were self‐reported by participants and cross‐checked against available medical records where possible; no formal comorbidity index (e.g., Charlson) was applied. Dyspnoea was recorded using the Medical Research Council (MRC) scale (Bestall et al. 1999). Functional mobility was assessed with the TUG test using the classic single‐task protocol (mean of three attempts from a 45–46 cm armchair, timing from the command ‘go’ to being seated again) (Podsiadlo and Richardson 1991). Quadriceps ultrasound was performed with a 5–10 MHz linear transducer in B‐mode (Katari et al. 2018), with the participant supine in the Semi‐Fowler position, knees extended and the lower limb in neutral rotation (Weinel et al. 2019); the transducer was positioned perpendicular to the limb at two‐thirds of the distance between the anterior superior iliac spine and the lateral femoral condyle (Silva et al. 2018). Rectus femoris (RF) echo intensity and thickness, and vastus intermedius (VI) thickness, were recorded; echo intensity was quantified by grayscale analysis (0–255) within a 50 × 50 mm square region of interest using ImageJ (NIH, Bethesda, MD) (Toledo et al. 2017; Parry et al. 2015; Grimm et al. 2013; Pillen and van Alfen 2011). Maximal inspiratory pressure (MIP) was assessed with a computerised device (KH2; POWERbreathe International Ltd., UK): seated with a nose clip, participants exhaled to residual volume and performed a maximal sustained inspiration (efforts < 1.5 s discarded); three to eight manoeuvres were performed until the between‐value variation was < 10%, and the highest value was recorded (Dias et al. 2013). All measurements were performed in duplicate.

### 2.4 Ethical Considerations

This study was approved by the authors' institutional Research Ethics Committee [committee name and approval number blinded for review], in accordance with Resolution 466/12 of the Brazilian National Health Council, and was registered on ClinicalTrials.gov [registration number blinded for review]. All participants provided written informed consent. The study was conducted in accordance with the Declaration of Helsinki.

### 2.5 Statistical Analysis

Descriptive statistics were expressed as mean ± standard deviation (SD) or median (interquartile range, IQR) according to distributional characteristics. Normality was assessed using the Shapiro–Wilk test (recommended for *n* < 30). Fall prevalence was expressed as a proportion with 95% Clopper–Pearson exact confidence intervals.

Between‐group comparisons (fallers vs. non‐fallers) used the Mann–Whitney *U* test for continuous variables and Fisher's exact test for categorical variables. Effect sizes were reported as Cohen's *d* (Cohen 1988) for normally distributed variables and rank‐biserial correlation (*r* = *Z*/√N) for Mann–Whitney tests. All estimates are unadjusted given the pilot sample size.

Discriminatory performance was evaluated by receiver operating characteristic (ROC) curve analysis. The area under the curve (AUC) quantifies discriminatory ability (0.5 = no discrimination; 0.7–0.8 = acceptable; 0.8–0.9 = excellent; > 0.9 = exceptional [26]). Bootstrap 95% CIs (2000 resamples) were calculated for each AUC; a variable was considered to show statistically significant discrimination when its 95% bootstrap CI excluded 0.5. The optimal clinical cut‐off was defined by the Youden index (*J* = sensitivity + specificity − 1). Positive and negative predictive values (PPV, NPV) and likelihood ratios (+LR, −LR) were calculated at the optimal cut‐off; post‐test probabilities were derived by Bayes' theorem. To assess overfitting, apparent AUCs and Youden‐derived operating characteristics were internally validated using Harrell's optimism‐correction bootstrap (2000 resamples): the direction of association for each variable was pre‐specified, optimal cut‐offs were re‐derived within each resample and applied to the original sample, and optimism (mean of resampled minus original performance) was subtracted from the apparent estimates. Spearman correlations (*ρ*) assessed associations between cumulative symptom months and total fall count. A sensitivity analysis restricted the discriminatory analysis to recurrent falls (≥ 2 falls, *n* = 7) versus non‐fallers (*n* = 14), motivated by the clinical relevance of identifying individuals with a higher fall burden; this analysis is exploratory given the limited statistical power afforded by the small subgroup size. Sample‐size estimation for the confirmatory study was performed post hoc using the Hanley–McNeil method (Hanley and McNeil 1982). Analyses were performed in Python 3 (SciPy 1.11; scikit‐learn 1.3) and JAMOVI v2.x (*α* = 0.05, two‐tailed). Given the exploratory, hypothesis‐generating design and the number of comparisons performed across diary variables and baseline measures, no formal correction for multiple comparisons (e.g., Bonferroni) was applied; reported *p*‐values should therefore be interpreted as descriptive of this pilot sample rather than as confirmatory hypothesis tests. Model calibration was not formally assessed; only discriminatory ability (AUC) was reported here, and calibration should be evaluated in an adequately powered confirmatory study.

## 3 Results

### 3.1 Sample Characteristics

Screening, enrolment, and completion of follow‐up are illustrated in the participant flow diagram (Figure 1). Diary data were complete for all participants who completed follow‐up; no imputation was required. Of 195 individuals screened, 165 were excluded and 30 were enrolled; after three losses to follow‐up, 27 completed 6‐month follow‐up (15 men, 55.6%; mean age 65.5 ± 9.4 years; BMI 25.7 ± 5.2 kg/m^2^). GOLD distribution: GOLD 1, 25.9%; GOLD 2, 44.4%; GOLD 3, 18.5%; GOLD 4, 11.1%. Mean FEV1% predicted was 48.5 ± 19.9%; FEV1/FVC was 0.57 ± 0.11. All were current or former smokers (100%). Systemic arterial hypertension and type 2 diabetes mellitus were each present in 33.3% of the participants. Median MRC dyspnoea scale was 2 (IQR 1–3). Full sample characteristics, stratified by fall status, are presented in Table 1.

**FIGURE 1:**
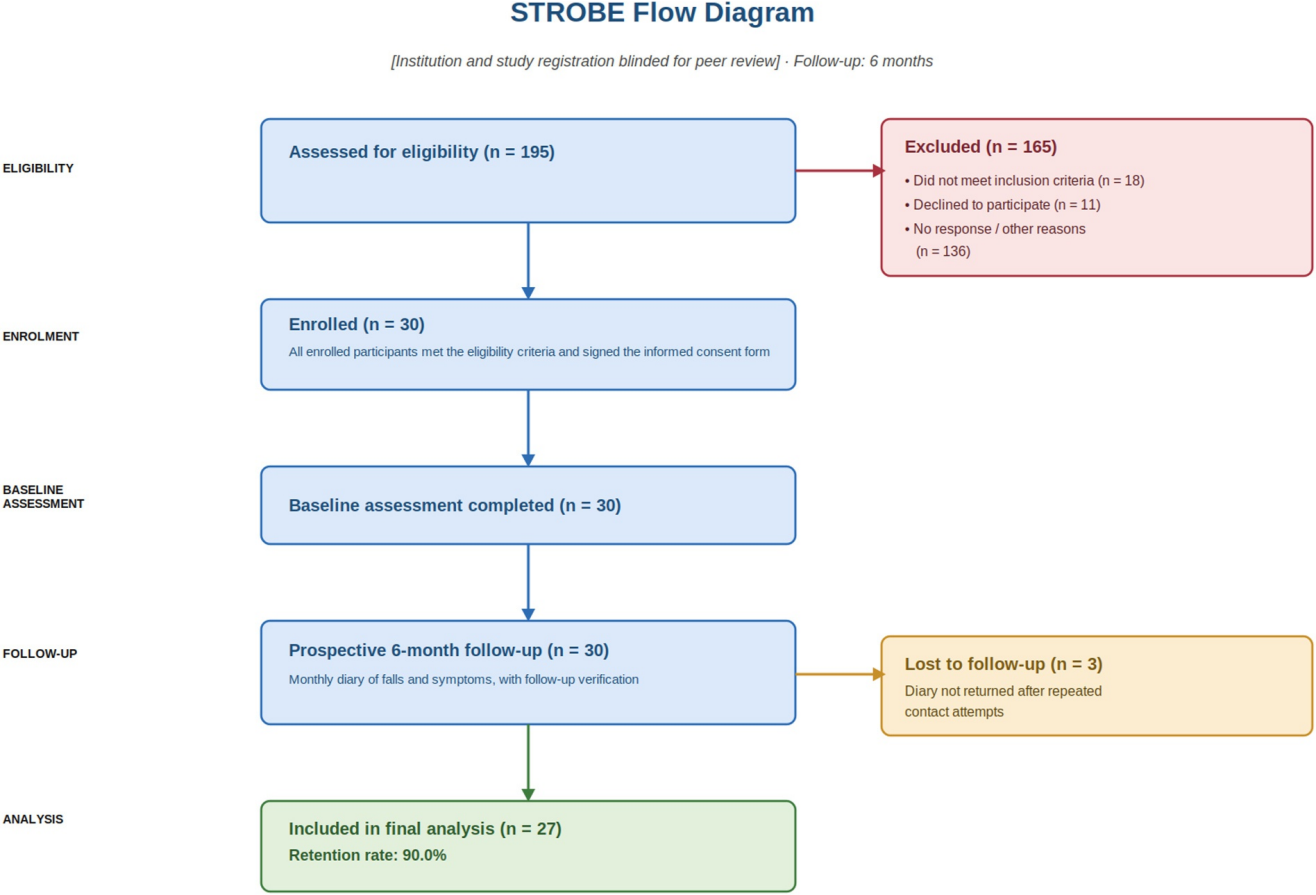
Participant flow diagram (STROBE). Of 195 individuals assessed for eligibility, 165 were excluded, 30 were enrolled, and 27 completed the 6‐month follow‐up.

### 3.2 Prevalence and Pattern of Falls

Thirteen patients (48.1%; 95% CI 28%–69%) experienced at least one fall, accumulating 23 falls in total. Distribution by fall frequency: 0 falls, 14 patients (51.9%); 1 fall, 6 patients (22.2%); 2 falls, 4 patients (14.8%); 3 falls, 3 patients (11.1%). Seven patients (25.9%) qualified as recurrent fallers (≥ 2 falls). Based on diary reports and monthly contact records, falls clustered predominantly in the first three months and were most commonly associated with dizziness or perceived lower limb weakness during postural transitions; individual fall counts and associated symptom‐months for all 13 fallers are provided in Supporting Information S3. No falls resulted in fractures requiring emergency hospitalisation.

### 3.3 Baseline Measures: Fallers Versus Non‐Fallers

None of the baseline physiological measures significantly differentiated fallers from non‐fallers (Table 1; all *p* > 0.05, except for FEV1/FVC). TUG time was numerically faster in fallers than in non‐fallers (9.52 ± 2.75 vs. 10.03 ± 2.39 s; *p* = 0.512). MIP (56.6 ± 25.9 vs. 53.3 ± 18.8 cmH_2_O; *p* = 0.903), quadriceps echogenicity (RF: *p* = 0.865; VI: *p* = 1.000), and VI thickness (8.79 ± 2.70 vs. 7.07 ± 4.24 mm; *p* = 0.132) also showed no significant between‐group differences. The FEV1/FVC ratio was paradoxically higher in fallers, a statistically significant difference (0.61 ± 0.09 vs. 0.53 ± 0.10; *p* = 0.041).

### 3.4 Diary Symptoms: Correlations With Fall Count

Spearman correlations between cumulative symptom months and total fall count are presented in Table 2. Months of dizziness showed the strongest association (*ρ* = 0.624; *p* = 0.001), followed by months of feeling unwell (*ρ* = 0.416; *p* = 0.031) and months of lower limb weakness (*ρ* = 0.401; *p* = 0.038). In Mann–Whitney U comparisons, fallers reported significantly more months of dizziness (1.38 ± 1.19 vs. 0.14 ± 0.36 months; *p* = 0.002; Cohen's *d* = 1.44; rank‐biserial *r* = 0.626) and more months of lower limb weakness (4.08 ± 1.55 vs. 2.21 ± 2.29 months; *p* = 0.034) than non‐fallers. In the recurrent‐falls subgroup (≥ 2 falls), months of dizziness remained significant (1.71 ± 1.38 vs. 0.14 ± 0.36 months; *p* = 0.004), while months of lower limb weakness did not reach significance (*p* = 0.088). Twelve of the fourteen non‐fallers reported less than 1 month of dizziness during the entire follow‐up, while more than two‐thirds of fallers reported at least 1 month, consistent with the high specificity (85.7%) of the ≥ 1‐month cut‐off.

**TABLE 2:** Longitudinal diary variables: Spearman correlations with fall count and Mann–Whitney U comparison (*n* = 27).

Diary variable	*ρ* (fall count)	95% CI for *ρ*	*p*	Fallers versus non‐fallers (mean ± SD)	Effect size (*r*)	*p* (MWU)
Months of dizziness	0.624	0.320, 0.811	0.001^**^	1.38 ± 1.19 versus 0.14 ± 0.36	*r* = 0.626	0.002^**^
Months of lower limb weakness	0.401	0.024, 0.678	0.038^*^	4.08 ± 1.55 versus 2.21 ± 2.29	*r* = 0.478	0.034^*^
Months of feeling unwell	0.416	0.043, 0.687	0.031^*^	2.38 ± 1.50 versus 1.36 ± 1.39	*r* = 0.401	0.075
Months of extra breathlessness	0.342	−0.044, 0.639	0.081	2.23 ± 1.92 versus 1.21 ± 1.37	*r* = 0.308	0.170
Months of difficulty sleeping	0.331	−0.056, 0.632	0.091	0.92 ± 1.19 versus 0.29 ± 0.61	*r* = 0.341	0.085
Months of difficulty rising	0.089	−0.301, 0.454	0.658	2.00 ± 1.96 versus 1.71 ± 2.09	*r* = 0.132	0.563
Months of difficulty walking	0.120	−0.273, 0.478	0.552	3.69 ± 2.18 versus 3.29 ± 2.23	*r* = 0.104	0.657

*Note: ρ*: Spearman correlation with total fall count; 95% CI calculated using Fisher's z‐transformation. MWU: Mann–Whitney U, effect size expressed as rank‐biserial correlation (*r*; positive values indicate higher values among fallers)—fallers (≥ 1 fall, *n* = 13) versus non‐fallers (*n* = 14). All estimates unadjusted (pilot study).

^*^ *p* < 0.05.

^**^ *p* < 0.01.

### 3.5 ROC Analysis and Internal Validation: Fall Discrimination

ROC curve results for all candidate variables are presented in Table 3 and illustrated in Figure 2. Months of dizziness showed the strongest discrimination (AUC = 0.813; 95% bootstrap CI [0.655–0.949]; *p* = 0.002). In 2000 bootstrap resamples, 92.4% yielded AUC > 0.70, 99.5% yielded AUC > 0.60, and 100% exceeded AUC 0.50. The Months of lower limb weakness showed the second‐strongest significant discrimination (AUC = 0.739; 95% CI [0.544–0.915]; *p* = 0.034; optimal cut‐off ≥ 2 months: sensitivity 92.3%, specificity 57.1%, *J* = 0.494). FEV1/FVC also showed acceptable discriminatory power (AUC = 0.734; *p* = 0.041; cut‐off ≥ 0.65; specificity 92.9%). All other candidate variables (TUG, MIP, and quadriceps echo intensity) showed AUC values not significantly different from chance (all *p* > 0.05).

**TABLE 3:** ROC analysis with internal validation and diagnostic performance: discrimination of fall occurrence (≥ 1 fall) (*n* = 27; all estimates unadjusted).

Predictor	AUC (95% CI)	Optimism‐corrected AUC^b^	Cut‐off	Sensitivity (95% CI)	Specificity (95% CI)	PPV	NPV	+LR	−LR	Youden J
Months of dizziness^a^	0.813 [0.66–0.95]	0.81	≥ 1 mo	69.2% [42.4–87.3]	85.7% [60.1–96.0]	81.8%	75.0%	4.84	0.36	0.549
Months of lower limb weakness^a^	0.739 [0.54–0.92]	0.74	≥ 2 mo	92.3% [66.7–98.6]	57.1% [32.6–78.6]	66.7%	88.9%	2.15	0.13	0.494
FEV1/FVC ratio^a^	0.734 [0.52–0.91]	0.73	≥ 0.65	53.8% [29.1–76.8]	92.9% [68.5–98.7]	87.5%	68.4%	7.54	0.50	0.467
Months of feeling unwell	0.701 [0.49–0.89]	—	≥ 2 mo	69.2% [42.4–87.3]	64.3% [38.8–83.7]	64.3%	69.2%	1.94	0.48	0.335
VI thickness (mm)	0.673 [0.44–0.88]	—	≥ 5 mm	100.0% [77.2–100.0]	35.7% [16.3–61.2]	59.1%	100.0%	1.56	0.00	0.357
TUG (s)	0.423 [0.21–0.66]	—	≥ 11.8 s	23.1% [8.2–50.3]	85.7% [60.1–96.0]	60.0%	54.5%	1.62	0.90	0.088
MIP (cmH_2_O)	0.484 [0.26–0.71]	—	≥ 36.8	92.3% [66.7–98.6]	28.6% [11.7–54.6]	54.5%	80.0%	1.29	0.27	0.209
RF echo intensity (AU)	0.478 [0.25–0.71]	—	≥ 50	100.0% [77.2–100.0]	14.3% [4.0–39.9]	52.0%	100.0%	1.17	0.00	0.143

*Note:* 95% CI, bootstrap (2000 resamples) for AUC, Wilson score interval for sensitivity/specificity. Youden *J* = sensitivity + specificity − 1.

Abbreviations: AU, arbitrary units; AUC, area under the ROC curve; +LR, positive likelihood ratio; −LR, negative likelihood ratio; MIP, maximal inspiratory pressure; NPV, negative predictive value; PPV, positive predictive value; RF, rectus femoris; TUG, Timed Up and Go; VI, vastus intermedius.

^a^ Statistically significant discriminatory power (*p* < 0.05).

^b^Optimism‐corrected AUC from Harrell's bootstrap (2000 resamples), reported for variables with significant discrimination; corrected sensitivity/specificity at the dizziness and weakness cut‐offs were 66%/83% and 88%/54%, respectively.

**FIGURE 2:**
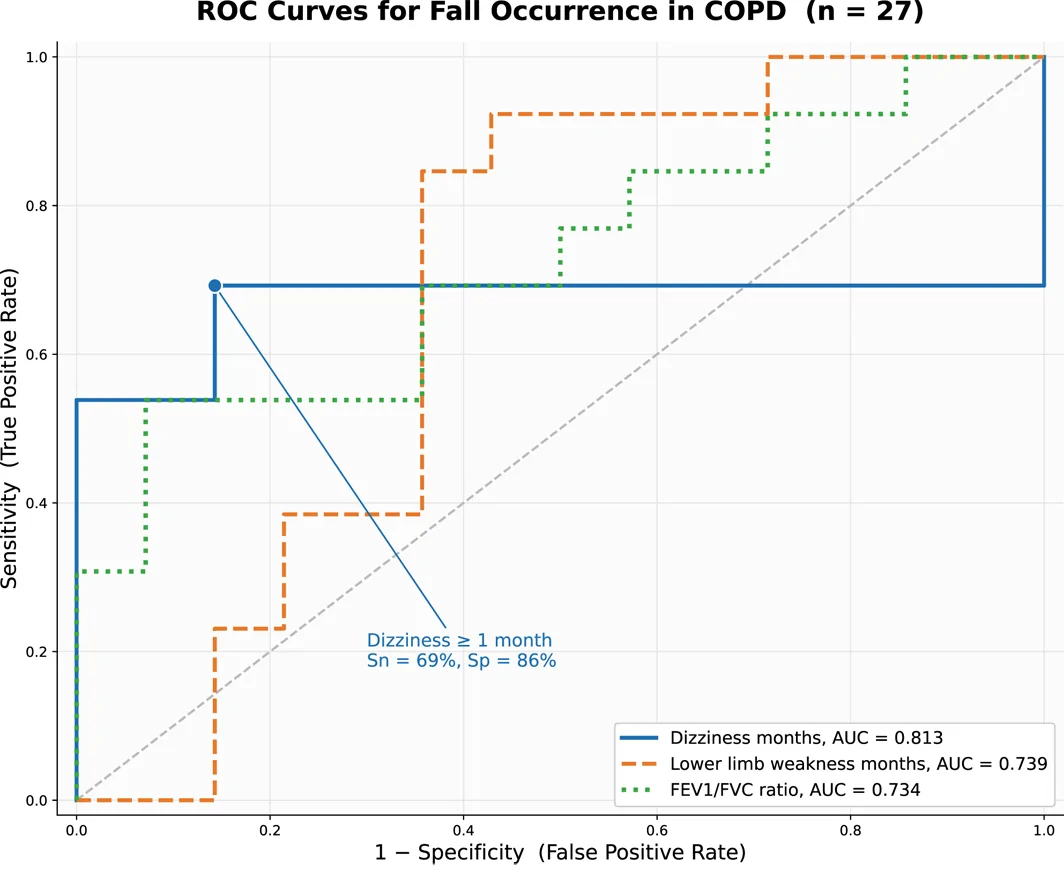
ROC curves for discriminating participants with versus without falls (≥ 1 fall during 6‐month follow‐up) using longitudinal diary variables and baseline physiological measures (*n* = 27). Months of dizziness (AUC = 0.813), months of lower limb weakness (AUC = 0.739), and the FEV1/FVC ratio (AUC = 0.734) were the only three variables with discriminatory power significantly greater than chance (*p* < 0.05; 2000 bootstrap resamples). The diagonal dashed line represents the null hypothesis (AUC = 0.50). ROC curves for the remaining variables are available in Figure S1.

Internal bootstrap validation (2000 resamples) indicated minimal optimism in discrimination: the optimism‐corrected AUC was 0.81 for months of dizziness (apparent 0.813), 0.74 for months of lower limb weakness (apparent 0.739), and 0.73 for FEV1/FVC (apparent 0.734). As expected, optimism was concentrated in the data‐driven cut‐offs: for the dizziness threshold (≥ 1 month), optimism‐corrected sensitivity/specificity were 66%/83% (apparent 69.2%/85.7%), and for the lower limb weakness threshold (≥ 2 months), 88%/54% (apparent 92.3%/57.1%). Discrimination therefore appears stable to internal resampling, whereas the operating characteristics of the thresholds require external confirmation. Based on the observed dizziness AUC (0.813) and the 48.1% fall prevalence, the Hanley–McNeil method (Hanley and McNeil 1982) estimated 77 participants for 80% power (*α* = 0.05, two‐tailed); allowing for 15%–20% attrition, a confirmatory sample of *N* ≥ 90 (approximately 100) was recommended (Table S1).

## 4 Discussion

### 4.1 Key Findings

The main finding of this pilot study is that dizziness and lower limb weakness, monitored prospectively through monthly home diaries, showed, in this pilot sample, greater apparent discriminatory capacity in this concurrent, within‐period analysis than the conventional baseline physiological assessments evaluated here. Months of dizziness yielded an AUC of 0.813, conventionally labelled ‘excellent’ discriminatory power (Hosmer et al. 2013); however, given the wide confidence interval and the absence of external validation, this label cannot be firmly claimed here and should be interpreted cautiously. Internal bootstrap validation indicated that this discrimination was minimally optimistic (optimism‐corrected AUC 0.81). These findings are preliminary and hypothesis‐generating, requiring confirmation in adequately powered studies.

### 4.2 Why Conventional Baseline Measures Did Not Discriminate

The inability of baseline assessments (TUG, MIP, and quadriceps ultrasound) to discriminate between participants who fell and those who did not replicate is a well‐documented phenomenon in the COPD literature. A prospective study of 99 patients found that TUG, the Berg Balance Scale, and grip strength did not predict falls during 12‐month follow‐up (Beauchamp et al. 2012), and data from a national survey confirmed that disease severity, hospitalisation history, and comorbidities collectively explained little variation in fall occurrence (Hakamy et al. 2018).

A mechanistic explanation lies in the episodic nature of falls. A fall results from a momentary mismatch between postural demand and postural capacity. Single‐visit assessments capture the stable characteristic but are structurally blind to the transient disturbance, which is precisely what the diary captures (McCrum et al. 2022; Ferreira et al. 2022). This does not imply that baseline physiological assessments are clinically useless; they remain valuable for characterising functional status and disease severity, but their standalone performance for discriminating participants with versus without falls appears limited in this and previous cohorts.

### 4.3 Mechanisms Underlying Dizziness as a Dominant Discriminator

Several mechanisms have been proposed in the literature to explain dizziness in individuals with COPD, including exercise‐induced arterial oxygen desaturation (patients with GOLD 2–3 often experience SpO_2_ reductions of 5–10% points during daily activities, which may acutely affect vestibular processing and cerebellar coordination) (Roig et al. 2011), medication‐induced haemodynamic instability (antihypertensives, diuretics, and antidiabetics can cause orthostatic hypotension and reduced baroreflex response, especially in older adults) (Tinetti et al. 2000), and vestibular or cerebellar effects of chronic hypoxia (prolonged low‐level hypoxia may affect Purkinje cells in the cerebellar vermis, impairing vestibulo‐ocular reflex modulation) (Menant et al. 2012). However, none of these mechanisms was directly assessed in the present study. The explanations above should therefore be interpreted as biologically plausible hypotheses drawn from the literature, rather than mechanisms demonstrated by our data, and warrant direct investigation in future studies.

### 4.4 Lower Limb Weakness: Complementary Discriminator

Lower limb weakness for ≥ 2 months was the second most discriminating variable (AUC = 0.739; *p* = 0.034), with high sensitivity (92.3%), a profile that would suit screening if confirmed in validated cohorts, though moderate specificity (57.1%) limits its stand‐alone use. The fact that diary‐recorded months of weakness discriminated fallers from non‐fallers while VI thickness by baseline ultrasound did not (*p* = 0.132) illustrates the limitation of static morphological measurements: muscle mass may be preserved while functional reserve is depleted by chronic fatigue and inflammation, a dissociation previously described in peripheral muscle studies in COPD (Robles et al. 2011).

### 4.5 Unexpected Finding: FEV1/FVC Ratio

An unexpected finding was the higher FEV1/FVC ratio observed among participants who experienced falls (0.61 ± 0.09 vs. 0.53 ± 0.10; *p* = 0.041). This result contrasts with the traditional expectation that more severe airflow obstruction would be associated with poorer functional status and greater risk of adverse outcomes. One possible explanation is that individuals with better‐preserved lung function may remain more physically active and therefore experience greater exposure to mobility‐related situations that increase opportunities for falls. However, physical activity was not measured in this study, and this interpretation remains speculative. Given the small sample size and the multifactorial nature of falls, this may also represent a chance finding reflecting random variation and unstable estimates. It should therefore be interpreted cautiously and confirmed in larger prospective studies.

### 4.6 Limitations

This study has nine main limitations. First, the sample (*n* = 27) was recruited from a single university referral centre [name blinded for review], limiting statistical power and generalisability; the small sample size increases the possibility of unstable effect estimates and wide confidence intervals, and all findings should be considered exploratory and hypothesis‐generating. Second, fall and symptom ascertainment relied on self‐report through a home diary consolidated by monthly structured contact; although designed to minimise recall bias, self‐reported events in COPD may diverge from healthcare records and be under‐reported (Grimwood et al. 2020), self‐report is susceptible to memory distortion and response bias (Anvari et al. 2023), and adherence to symptom diaries in COPD can vary (Lee et al. 2017); future studies should complement diary recording with accelerometry‐based fall detection (Ferreira et al. 2022). Third, dizziness and lower limb weakness were recorded as monthly binary indicators rather than with validated intensity scales such as the Dizziness Handicap Inventory; symptom severity, duration, and frequency were therefore not captured, which may have reduced the precision of the observed associations. Fourth, multivariate adjustment was not feasible with *n* = 27; in particular, factors known to influence both dizziness and falls, including prior fall history, orthostatic hypotension, vestibular disorders, peripheral neuropathy, visual impairment, and polypharmacy, were not statistically controlled, and their potential confounding effect cannot be excluded. Fifth, the recurrent‐falls subgroup (*n* = 7) has very low statistical power and its results should be considered hypothesis‐generating only. Sixth, the 6‐month follow‐up may not capture seasonal variation; a 12‐month follow‐up is planned for the confirmatory study. Seventh, although the ROC cut‐offs were derived and evaluated on the same dataset, internal bootstrap validation showed that discrimination was minimally optimistic (optimism‐corrected AUC ≈ apparent), with optimism confined to the operating characteristics of the data‐driven thresholds; nonetheless, external validation in an independent cohort remains necessary before these thresholds can be considered for clinical use. Eighth, because symptoms were consolidated on a monthly basis, it was not possible to determine whether dizziness or lower limb weakness occurred immediately before a fall or several weeks earlier; the temporal relationship should therefore be interpreted cautiously. More importantly, symptom‐months and fall counts were both summed over the entire 6‐month window, so symptom‐months occurring after a participant's first fall could still contribute to that participant's cumulative symptom‐month value; the dataset does not contain a month‐by‐month or date‐level record that would allow symptom exposure to be censored at the time of each participant's first fall. Consequently, the reported associations should be understood as concurrent, within‐period associations rather than as evidence that symptom monitoring temporally precedes and predicts subsequent falls; this constraint is the reason discriminatory (rather than predictive) language is used throughout this manuscript, pending a study design that captures fall and symptom timing at the month level. Ninth, diary data were complete for all participants who completed follow‐up, with no missing entries requiring imputation; however, adherence among the three participants lost to follow‐up could not be assessed.

Regarding external validity, this sample was recruited from an urban university referral centre in north‐east Brazil. Generalisation to primary care settings, rural populations, or health systems outside Brazil requires caution.

## 5 Implications for Physiotherapy Practice

Although these findings are promising, they should be interpreted cautiously given the pilot nature of the study, the small sample size, and the absence of external validation. With this caveat, monthly home diary monitoring of dizziness and lower limb weakness may eventually complement existing fall‐risk assessment strategies in COPD, such as functional assessment, medication review, balance training, and pulmonary rehabilitation, pending confirmation in adequately powered prospective studies. If confirmed in larger studies, structured symptom diaries could be integrated into the routine physiotherapy follow‐up of individuals with COPD, where such follow‐up is available: the monthly schedule is low‐cost, requires no additional equipment, and can be administered by telephone. The near absence of dizziness in non‐fallers and the high sensitivity of lower limb weakness for ≥ 2 months suggest these symptoms may, if confirmed in studies with month‐level temporal resolution, help flag patients to prompt closer physiotherapy review, medication reconciliation, and targeted balance rehabilitation. Such multicomponent approaches, combining medication review, balance training (McCrum et al. 2022), and pulmonary rehabilitation (Dias et al. 2013), have established precedents in falls prevention and COPD care, providing a framework into which diary‐triggered review could fit. These observations are preliminary and should not be implemented into clinical practice without confirmation in adequately powered prospective studies.

## 6 Conclusion

In this exploratory pilot study, longitudinal diary monitoring of dizziness and lower limb weakness discriminated participants who experienced falls more effectively than single‐visit baseline physiological measures (TUG, MIP, and quadriceps ultrasound). The central implication is that the cumulative burden of symptoms over the follow‐up period, rather than static physiological status, is more strongly associated with falls in COPD; because symptom‐months and falls were both accrued over the same 6‐month window, this association should not yet be read as evidence that symptom monitoring temporally precedes and predicts falls. These findings provide preliminary, hypothesis‐generating evidence supporting the potential relevance of longitudinal symptom monitoring in fall‐risk assessment among individuals with COPD. Confirmation in adequately powered prospective studies is required before clinical application. A multicentre confirmatory study with extended follow‐up and objective fall‐detection methods is warranted, with sample size estimated in Table S1.

## Author Contributions

**Danielle Ferreira de Santana Silva:** conceptualization, data collection, statistical analysis, manuscript writing (lead). **Bianca Maria Santos Alcântara:** data collection. **Jefferson Ricardo Santos da Silva:** data management and digital instruments. **Rafaela Pedrosa:** data collection. **Eduardo Eriko Tenório de França:** critical revision. **José Heriston de Morais Lima:** conceptualization, supervision, critical revision. All authors reviewed and approved the final version and agree to be accountable for all aspects of the work.

## Funding

This work was carried out within the project “Acute effects of inspiratory muscle training with different loads on peripheral muscle metabolism and autonomic response in patients with COPD: a randomized crossover trial”, supported by the Brazilian National Council for Scientific and Technological Development (CNPq/MCTI Call No. 10/2023—Band A, Emerging Groups; process No. 404976/2023‐9). The funder played no role in study design, data collection, analysis, interpretation, or manuscript preparation.

## Ethics Statement

This study was approved by the Research Ethics Committee of the Federal University of Paraíba (CEP/UFPB)—approval number 6.669.065, in accordance with Resolution 466/12 of the Brazilian National Health Council and the Declaration of Helsinki.

## Consent

All participants provided written informed consent prior to enrolment, in accordance with Resolution 466/12 of the Brazilian National Health Council.

## Conflicts of Interest

The authors declare no conflicts of interest.

## Supporting information


Supporting Information S1

Supporting Information S2

Supporting Information S3

**Figure S1:** Receiver operating characteristic (ROC) curves for baseline physiological measures without statistically significant discriminatory performance for fall occurrence: Timed Up and Go (TUG, AUC = 0.423), maximal inspiratory pressure (MIP, AUC = 0.484), and rectus femoris echo intensity (AUC = 0.478). The diagonal dashed line represents no discrimination (AUC = 0.50).

**Table S1:** Sample‐size estimation for the confirmatory study (primary discriminating variable: months of dizziness).

## Data Availability

The dataset supporting the conclusions of this article is available from the corresponding author upon reasonable request.
