# Supplementary material for: Dizziness, Lower Limb Weakness, and Falls in COPD: A Prospective Longitudinal Pilot Study Using Monthly Home Diaries
Source: Physiother Res Int. 2026 Oct 22;31(4):e70363. doi: 10.1002/pri.70363 (PMC13688226; doi:10.1002/pri.70363)
Supplement: Supplementary file 1 [file PRI-31-e70363-s001.docx]

# Supplementary File S2. Per-participant fall counts and cumulative symptom-months among fallers

Individual data for the 13 participants who experienced at least one fall during the six-month follow-up (n = 27). Values are the cumulative number of months (0–6) in which each symptom was recorded in the monthly home diary. Participants are anonymised (F01–F13) and ordered by fall count. Seven participants were recurrent fallers (≥2 falls).

| Participant | Falls (6 mo) | Dizziness | LL weakness | Feeling unwell | Extra breathlessness | Difficulty sleeping | Difficulty rising | Difficulty walking |
| --- | --- | --- | --- | --- | --- | --- | --- | --- |
| F01 | 3 | 4 | 6 | 4 | 5 | 4 | 2 | 6 |
| F02 | 3 | 2 | 3 | 2 | 3 | 2 | 2 | 5 |
| F03 | 3 | 0 | 5 | 5 | 3 | 0 | 0 | 5 |
| F04 | 2 | 2 | 4 | 3 | 3 | 0 | 5 | 0 |
| F05 | 2 | 2 | 4 | 2 | 1 | 0 | 1 | 5 |
| F06 | 2 | 2 | 2 | 2 | 0 | 0 | 0 | 2 |
| F07 | 2 | 0 | 4 | 1 | 2 | 1 | 2 | 3 |
| F08 | 1 | 2 | 6 | 4 | 1 | 0 | 0 | 5 |
| F09 | 1 | 2 | 1 | 2 | 5 | 1 | 1 | 2 |
| F10 | 1 | 1 | 5 | 4 | 5 | 2 | 5 | 6 |
| F11 | 1 | 1 | 3 | 0 | 0 | 1 | 0 | 0 |
| F12 | 1 | 0 | 6 | 1 | 0 | 0 | 3 | 6 |
| F13 | 1 | 0 | 4 | 1 | 1 | 1 | 5 | 3 |

*All symptom values are months (0–6). LL weakness: lower limb weakness. Recurrent fallers (F01–F07) accumulated ≥2 falls; single fallers (F08–F13) had exactly one fall. Dizziness ≥1 month was present in 9 of 13 fallers (69.2%), consistent with the ≥1-month cut-off reported in Table 3.*
