# Supplementary material for: Dizziness, Lower Limb Weakness, and Falls in COPD: A Prospective Longitudinal Pilot Study Using Monthly Home Diaries
Source: Physiother Res Int. 2026 Oct 22;31(4):e70363. doi: 10.1002/pri.70363 (PMC13688226; doi:10.1002/pri.70363)
Supplement: Supplementary file 2 [file PRI-31-e70363-s002.docx]

**Supplementary Figure S1.** Receiver operating characteristic (ROC) curves for baseline physiological measures without statistically significant discriminatory performance for fall occurrence: Timed Up and Go (TUG, AUC = 0.423), maximal inspiratory pressure (MIP, AUC = 0.484), and rectus femoris echo intensity (AUC = 0.478). The diagonal dashed line represents no discrimination (AUC = 0.50).
