# Supplementary material for: Dizziness, Lower Limb Weakness, and Falls in COPD: A Prospective Longitudinal Pilot Study Using Monthly Home Diaries
Source: Physiother Res Int. 2026 Oct 22;31(4):e70363. doi: 10.1002/pri.70363 (PMC13688226; doi:10.1002/pri.70363)
Supplement: Supplementary file 3 [file PRI-31-e70363-s003.docx]

# Supplementary File S1. Standardised symptom reference card and home-diary instrument

This file reproduces the standardised instrument provided to each participant at enrolment and used to consolidate the monthly home diary (LAFIPCARE, Federal University of Paraíba). It comprises (a) the fall definition and daily fall-registration sheet and (b) the monthly symptom questions (“reference card”) that operationalised each of the seven monitored symptoms in plain language. The original wording (Portuguese, as administered) is given together with an English translation. Symptoms were recorded as present/absent each month; the cumulative number of months with each symptom (range 0–6) was used in the analysis.

## Part A — Fall definition and daily fall register (diary cover instructions)

**Original (Portuguese):** “É importante que você saiba o que é considerado uma queda. Uma queda não é apenas cair no chão. Qualquer momento em que você perdeu o equilíbrio, mesmo que tenha conseguido se segurar em algo ou em alguém para não cair completamente, deve ser registrado.”

**English translation:** “It is important to know what counts as a fall. A fall is not only landing on the ground. Any moment in which you lost your balance — even if you managed to hold on to something or someone to avoid falling completely — must be recorded.”

Each day, the participant recorded whether a fall or loss of balance occurred and, if so, completed the following fields (daily register, days 1–31 per month):

| Field (Portuguese) | Field (English) |
| --- | --- |
| Caiu ou desequilibrou? (Sim/Não) | Fell or lost balance? (Yes/No) |
| Onde aconteceu? | Where did it happen? |
| Machucou? (Sim/Não) | Any injury? (Yes/No) |
| O que aconteceu? | What happened? (brief description) |
| Procurou atendimento médico ou ajuda após a queda? (Sim/Não) | Sought medical care or help after the fall? (Yes/No) |

## Part B — Monthly symptom reference card (seven monitored symptoms)

Each month, a trained physiotherapist administered the standardised questions below (by telephone/messaging) to confirm and consolidate the diary entries. Each symptom was scored as present or absent for that month.

| # | Symptom (as reported in the manuscript) | Question as administered (Portuguese) | English translation | Response |
| --- | --- | --- | --- | --- |
| 1 | Dizziness or vertigo | Você sente tontura ou desequilíbrio com frequência? | Do you frequently feel dizziness or imbalance? | Yes / No |
| 2 | Lower limb weakness | Você sente fraqueza nas pernas? | Do you feel weakness in your legs? | Yes / No |
| 3 | Difficulty rising from a chair | Você tem dificuldade para levantar-se de uma cadeira sem apoio? | Do you have difficulty rising from a chair without support? | Yes / No |
| 4 | Difficulty walking | Você tem dificuldade para caminhar em superfícies irregulares? | Do you have difficulty walking on uneven surfaces? | Yes / No |
| 5 | Feeling generally unwell | Você adoeceu este mês? | Did you feel unwell / become ill this month? | Yes / No |
| 6 | Extra breathlessness beyond usual | Teve mais falta de ar do que o normal? | Did you have more breathlessness than usual? | Yes / No |
| 7 | Difficulty sleeping | Você tem dificuldade para dormir devido à falta de ar? | Do you have difficulty sleeping because of breathlessness? | Yes / No |

An open item was also included — “Sentiu tontura, fraqueza ou outros sintomas relevantes que não foram mencionados aqui? (Sim/Não; Qual?)” / “Did you feel dizziness, weakness, or any other relevant symptom not listed here? (Yes/No; which?)” — to capture symptoms outside the predefined list.

## Part C — Recording and scoring

Symptoms were recorded on a monthly, binary basis (present/absent). For each participant and each symptom, the number of months in which the symptom was present (0–6) was summed to yield the cumulative symptom-months used in the correlation and ROC analyses. No validated intensity scale (e.g., Dizziness Handicap Inventory) was applied; severity, duration within a month, and exact timing relative to falls were therefore not captured (see Limitations).

*Instrument developed for this study by the LAFIPCARE research team (Federal University of Paraíba). To our knowledge, no COPD-specific validated definitions exist for these items; they were treated as pragmatic, study-specific descriptors. Home diary and monthly questions correspond to Appendices C and D of the study protocol.*
