# Supplementary material for: Dizziness, Lower Limb Weakness, and Falls in COPD: A Prospective Longitudinal Pilot Study Using Monthly Home Diaries
Source: Physiother Res Int. 2026 Oct 22;31(4):e70363. doi: 10.1002/pri.70363 (PMC13688226; doi:10.1002/pri.70363)
Supplement: Supplementary file 4 [file PRI-31-e70363-s004.docx]

**Supplementary Table S1.** Sample-size estimation for the confirmatory study (primary discriminating variable: months of dizziness)

| **Parameter / Assumption** | **Value** | **Source** |
| --- | --- | --- |
| Target AUC (primary variable) | 0.813 | This pilot study |
| Null hypothesis (H₀: no discrimination) | AUC = 0.50 | Default null ROC |
| Significance level (α, two-tailed) | 0.05 | Conventional |
| Statistical power (1 − β) | 80% | Conventional minimum |
| Prevalence of falls (assumed) | 48.1% | This pilot study |
| Total N (Hanley–McNeil, prevalence-adjusted) | 77 | Hanley & McNeil (27) |
| Convergent proportion-based estimate | ≈81 | 30% prevalence, 95% CI, 10% margin |
| Recommended confirmatory sample (15–20% dropout) | N ≥ 90 (≈100) | Final recommendation |

*Sample size estimated primarily by the Hanley–McNeil method (27), adapted for unequal group sizes per observed fall prevalence; a proportion-based calculation converged on a similar range. AUC: area under the ROC curve.*
