# Supplementary material for: Dizziness, Lower Limb Weakness, and Falls in COPD: A Prospective Longitudinal Pilot Study Using Monthly Home Diaries
Source: Physiother Res Int. 2026 Oct 22;31(4):e70363. doi: 10.1002/pri.70363 (PMC13688226; doi:10.1002/pri.70363)
Supplement: Supplementary file 5 [file PRI-31-e70363-s005.docx]

**Supplementary File S1. Standardised symptom reference card and home-diary instrument.**

This file contains the fall definition and daily fall-registration sheet, together with the monthly symptom questions used to assess the seven monitored symptoms. The original Portuguese version and an English translation are provided. Symptoms were recorded as present/absent each month, and the cumulative number of months with each symptom (range 0–6) was used in the analysis.

**Supplementary File S2. Per-participant fall counts and cumulative symptom-months among fallers.**

Individual data for the 13 participants who experienced at least one fall during the six-month follow-up (n = 13; 23 falls). Values represent the cumulative number of months (0–6) in which each symptom was recorded in the monthly home diary. Participants are anonymised (F01–F13) and ordered by fall count. Seven participants were recurrent fallers (≥2 falls).
